# Use of the FHTHWA Index as a Novel Approach for Predicting the Incidence of Diabetes in a Japanese Population Without Diabetes: Data Analysis Study

**DOI:** 10.2196/64992

**Published:** 2025-01-28

**Authors:** Jiao Wang, Jianrong Chen, Ying Liu, Jixiong Xu

**Affiliations:** 1 Department of Endocrinology and Metabolism, The First Affiliated Hospital, Jiangxi Medical College, Nanchang University, Nanchang, China; 2 Jiangxi Clinical Research Center for Endocrine and Metabolic Disease, Nanchang, China; 3 Jiangxi Branch of National Clinical Research Center for Metabolic Disease, Nanchang, China

**Keywords:** prediction, diabetes, risk, index, population without diabetes

## Abstract

**Background:**

Many tools have been developed to predict the risk of diabetes in a population without diabetes; however, these tools have shortcomings that include the omission of race, inclusion of variables that are not readily available to patients, and low sensitivity or specificity.

**Objective:**

We aimed to develop and validate an easy, systematic index for predicting diabetes risk in the Asian population.

**Methods:**

We collected the data from the NAGALA (NAfld [nonalcoholic fatty liver disease] in the Gifu Area, Longitudinal Analysis) database. The least absolute shrinkage and selection operator model was used to select potentially relevant features. Multiple Cox proportional hazard analysis was used to develop a model based on the training set.

**Results:**

The final study population of 15464 participants had a mean age of 42 (range 18-79) years; 54.5% (8430) were men. The mean follow-up duration was 6.05 (SD 3.78) years. A total of 373 (2.41%) participants showed progression to diabetes during the follow-up period. Then, we established a novel parameter (the FHTHWA index), to evaluate the incidence of diabetes in a population without diabetes, comprising 6 parameters based on the training set. After multivariable adjustment, individuals in tertile 3 had a significantly higher rate of diabetes compared with those in tertile 1 (hazard ratio 32.141, 95% CI 11.545‐89.476). Time receiver operating characteristic curve analyses showed that the FHTHWA index had high accuracy, with the area under the curve value being around 0.9 during the more than 12 years of follow-up.

**Conclusions:**

This research successfully developed a diabetes risk assessment index tailored for the Japanese population by utilizing an extensive dataset and a wide range of indices. By categorizing the diabetes risk levels among Japanese individuals, this study offers a novel predictive tool for identifying potential patients, while also delivering valuable insights into diabetes prevention strategies for the healthy Japanese populace.

## Introduction

The increasing number of patients with diabetes mellitus represents an escalating challenge worldwide [1,2]. Based on the recently published International Diabetes Federation (IDF) Diabetes Atlas, the recorded worldwide incidence of diabetes among the adult population (20‐79 years) in 2021 was 10.5%, while the prevalence in Southeast Asia was 8.7% [3]. It is estimated that by the year 2045, the global prevalence of individuals living with diabetes will increase to an estimated 783 million, corresponding to a prevalence rate of approximately 12.2% [3], which indicates the urgent need for global diabetes prevention and control. Given the significant prevalence of patients with impaired fasting glucose or undiagnosed diabetes, it is imperative to prioritize the early screening and detection in these patients to mitigate the morbidity associated with diabetes, minimize healthcare expenditures, and prevent a decline in the quality of life [2,4].

Diabetes mellitus is a metabolic disorder, affecting both glucose and lipid metabolism [5,6]. Recent studies have investigated the correlation between glucose and lipid-related indexes and the prevalence of diabetes, including the triglyceride-glucose (TyG) index, metabolic score for insulin resistance (METS-IR), triglyceride to high-density lipoprotein cholesterol ratio (TG/HDL-C), and total cholesterol to high-density lipoprotein cholesterol ratio (TC/HDL-C) [7-9]. However, the accuracy of these indexes remains limited, and better prognostication indexes need to be found. Meanwhile, certain researchers have redirected their attention towards employing computational methods in order to predict diabetes at an early stage with greater precision [10-14]. The diabetes risk test created by the American Diabetes Association (ADA) serves as a screening tool designed to categorize individuals at a heightened risk within the community to enhance awareness regarding modifiable risk factors and promote the adoption of a healthy lifestyle [15]. Bang et al [16] developed a new screening score using the National Health and Nutrition Examination Survey (NHANES) and a combined cohort of two community studies However, the utilization of diabetes prediction and risk assessment models has been limited due to their predominantly designed focus on White populations. Specifically, there is a scarcity of scoring systems tailored to Japanese populations.

Therefore, in this study, we aimed to develop and validate an easy, systematic index for predicting the diabetes risk in the Asian population using glucose and lipid metabolism parameters and anthropometric parameters, which will enable the clinician or lay persons to assess their own risk of undiagnosed diabetes.

## Methods

### Study Population

The study participants were selected from the NAGALA (NAfld [nonalcoholic fatty liver disease] in the Gifu Area, Longitudinal Analysis) database—a human dock that serves as a repository for detecting chronic diseases and their linked risk factors, with the aim of advancing public health initiatives. The cohort has been comprehensively delineated in a prior investigation undertaken by Okamura et al [17]. The NAGALA cohort was initiated in 1994, encompassing individuals who underwent health examinations at Murakami Memorial Hospital. As a significant portion of the hospital’s screening participants, approximately 60%, return for annual or biennial check-ups, the NAGALA team implemented an extended follow-up study to monitor the emergence of diabetes and nonalcoholic fatty liver disease in this population over time. Furthermore, the available dataset relevant to the investigation cohort has been made accessible on the Dryad public database [18] to facilitate the sharing of data.

The present study incorporated the data of 20,944 individuals who were enrolled in the NAGALA cohort between 1994 and 2016. After the further exclusion of participants who were diagnosed with diabetes, impaired fasting glucose, liver disease, excessive drinking, missing data on potential covariates, baseline medication, and unexplained withdrawal from the survey, a total of 15,464 participants were included as the analytical sample.

### Ethical Considerations

The data used in this study were obtained from publicly available online databases. As these databases consist of deidentified and aggregated data, they do not involve human subjects or animals. All data were collected from publicly available sources; therefore, ethical approval was not required. Therefore, ethical review and approval were not required for this study in accordance with the ethical standards of the institutional and national research committees, as well as with the 1964 Helsinki Declaration and its later amendments or comparable ethical standards.

### Measurement

Demographic data, examination and laboratory test results, medical history, and prescription medication information were used from the NAGALA data. The laboratory test levels included alanine aminotransferase (ALT), aspartate aminotransferase (AST), gamma-glutamyl transferase (GGT), total cholesterol (TC), triglyceride (TG), fasting plasma glucose (FPG), high-density lipoprotein cholesterol (HDL-C), and low-density lipoprotein cholesterol (LDL-C). The examination and laboratory tests included the evaluation of the BMI, systolic blood pressure (SBP), diastolic blood pressure (DBP), and waist circumference (WC). Regarding exercise behaviors, the participants’ frequency of engaging in physical exercise per week was evaluated, with the criterion for the habit of exercise being defined as more than once per week. Alcohol consumption was categorized as heavy, moderate, light, and none, in line with the quantity and nature of participants’ weekly alcohol intake over the course of the preceding month [19]. Smoking status was categorized as current smoker, past smoker, and never smoker.

TyG index was calculated as follows: TyG index = Ln [fasting TGs (mg/dL) × fasting glucose (mg/dL) / 2] [20].

TG/HDL-C was calculated as follows: TG/HDL-C ratio = TG/HDL-C [21].

METS-IR was calculated as follows: METS-IR = Ln ((2 × FPG) + TG) × BMI) / (Ln (HDL-C)) [22].

Gastroenterologists made a diagnosis of fatty liver by evaluating specific characteristics observed on abdominal Doppler ultrasonography; these characteristics included vascular blurring, deep attenuation, liver brightness, and hepatorenal echo contrast [23].

The diagnosis of diabetes mellitus was established using the criteria set forth by the ADA. Participants were deemed to have diabetes if their HbA_1c_ measurements were ≥6.5% or if their FPG levels were ≥7 mmol/L during the follow-up period. Furthermore, the analysis incorporated the participant’s self-reported diabetes diagnosis during the follow-up period.

### Statistical Analysis

If the continuous variables followed a normal distribution, they were represented as the mean and the standard deviation. The variables exhibiting nonnormal distribution were presented as medians accompanied by their respective minimum and maximum values. To evaluate variations between the two groups, the statistical analysis employed the Pearson chi-square test for categorical variables, while the Mann-Whitney *U* test or Student *t*-test was utilized for continuous variables. One-way ANOVA was used to compare several groups of data that followed a normal distribution, while the Kruskal-Wallis test was used to analyze data that did not conform to a normal distribution.

A total of 15464 participants were randomly assigned to the training (n=9280, 60%) and internal validation (n=6184, 40%) sets. To establish the index for predicting the incidence of diabetes, univariate Cox regression was applied to detect the variables related to diabetes in the training set. Subsequently, the Cox regression model incorporating the least absolute shrinkage and selection operator (LASSO) was employed to ascertain the coefficients for constructing the model by utilizing an optimal log λ value. The index was established with the following formula: risk score = value of factor 1 × β1+ value of factor 2 × β2+…value of factor n × βn (β was the weighted coefficient of each factor).

Furthermore, an analysis of the receiver operating characteristic (ROC) curve was conducted, and areas under the curve (AUCs) were carried out to assess the predictive value of fasting stress hyperglycemia ratio, FPG, and HbA_1c_ in relation to the risk of diabetes.

The patients were divided into three groups (tertile 1: <33rd percentile, tertile 2: ≥33rd to 67th percentile, and tertile 3: ≥67th percentile) according to the novel index tertiles. Cox proportional hazards regression models were employed to assess the link between the index and the incidence of diabetes. The models included several covariates, namely sex, alcohol consumption, smoking status, habit of exercise, BMI, fatty liver, ALT, AST, GGT, TC, SBP, DBP, TyG, TG/HDL-C ratio, and METS-IR.

The statistical analyses were conducted using SPSS version 23.0 (SPSS Inc.) and R version 3.6.0 (R Foundation for Statistical Computing). A two-sided *P*-value <.05 was deemed to be statistically significant.

## Results

### Study Population

The current investigation encompassed 15,464 diabetes-free participants, with a mean age of 42 (range 18-79) years, 8430 (54.5%) men and 7034 (45.5%) women, and a mean follow-up of 6.05 (SD 3.7) years. A 6-to-4 ratio was used to randomly allocate patients into the training (n=9280) and validation (n=6184) sets. Table S1 in Multimedia Appendix 1 displays the variations in baseline characteristics of the study population in both the training set and validation set.

### Establishment of the Diabetes Incidence Index

A univariate analysis was conducted on the training dataset in order to ascertain the variables that were correlated with the occurrence of diabetes. Eight significant variables, namely age, BMI, WC, HDL-C, TC, TG, HbA_1c_, and FPG, were employed as predictive factors for progressing diabetes (*P*<.05; see Table S2 in Multimedia Appendix 1). The 8 clinical features were subsequently included in the LASSO regression analysis, which was performed for 1000 bootstrap iterations. From this analysis, 4 features were identified with nonzero coefficients and a minimum lambda value (Figure S1 in Multimedia Appendix 1). The findings of multivariate Cox regression analysis indicate that age, WC, HDL-C, TG, HbA_1c_, and FPG were identified as independent risk factors (Table S3 in Multimedia Appendix 1).

To develop a risk index, we constructed a clinical feature-based risk signature derived from the results of a multivariate Cox regression analysis. The risk score was calculated as follows:

[(FPG) × 1.525] – [(HDL-C) × 0.454] + [(TG) × 0.216] + [(HbA_1c_) × 3.298] + [(WC) × 0.044] + [(age) × 0.016].

We defined this new parameter combined with FPG, HDL-C, TG, HbA_1c_, WC, and age as the FHTHWA index based on the initial letter of each variable.

### Baseline Characteristics

The baseline characteristics of participants were examined based on the FHTHWA index in both the training and validation sets.

For the training set, patients with increased FHTHWA index levels tended to be of advanced age and displayed characteristics indicative of fatty liver, with higher BMI, WC, ALT, AST, body weight, GGT, TC, TG, HbA_1c_, FPG, SBP, DBP, alcohol consumption, smoking, TG/HDLC ratio, TyG index, and METS-IR, as well as low HDL-C levels (Table 1; all *P*<.01).

**Table 1.** Baseline characteristics of the study population by tertiles of the FHTHWA index in the training set.

Characteristics	Tertile 1 (n=3093)	Tertile 2 (n=3093)	Tertile 3 (n=3094)	*P* value
Sex, n (%)				<.001
Male	1106 (11.92)	1808 (19.48)	2174 (23.43)	<.001
Female	1987 (21.41)	1285 (13.85)	920 (9.91)	<.001
Age, years, mean (range)	40 (18-69)	43 (21-74)	47 (19-79)	<.001
BMI, kg/m^2^, mean (SD)	20.52 (2.36)	21.88 (2.70)	23.93 (3.28)	<.001
Waist circumference, cm, mean (SD)	70.94 (7.21)	76.36 (7.61)	82.21 (8.59)	<.001
Alanine aminotransferase, IU/L, mean (range)	15 (4-141)	16 (4-129)	21 (4-149)	<.001
Aspartate aminotransferase, IU/L, mean (range)	16 (7-98)	17 (3-150)	19 (6-94)	<.001
Body weight, kg, mean (SD)	54.70 (9.03)	60.31 (10.29)	66.77 (11.98)	<.001
Gamma-glutamyl transferase, IU/L, mean (range)	12 (4-248)	15 (5-399)	19 (3-259)	<.001
High-density lipoprotein cholesterol, mmol/L, mean (range)	1.58 (0.55-3.49)	1.42 (0-3.21)	1.24 (0-3.05)	<.001
Total cholesterol, mmol/L, mean (SD)	4.86 (0.80)	5.11 (0.83)	5.38 (0.87)	<.001
Triglyceride, mmol/L, mean (range)	0.55 (0.07-3.67)	0.73 (0.11-6.75)	1.03 (0.12-7.69)	<.001
Hemoglobin A1C, %, mean (SD)	4.89 (0.23)	5.15 (0.21)	5.46 (0.24)	<.001
Fasting plasma glucose, mmol/L, mean (SD)	4.83 (0.32)	5.15 (0.31)	5.49 (0.32)	<.001
Systolic blood pressure, mmHg, mean (SD)	108.47 (13.36)	114.51 (14.25)	120.71 (14.80)	<.001
Diastolic blood pressure, mmHg, mean SD	67.60 (9.42)	71.41 (10.14)	75.94 (10.28)	<.001
Fatty liver, n (%)				<.001
Yes	118 (1.27)	394 (4.25)	1134 (12.22)	
No	2975 (32.06)	2699 (29.08)	1960 (21.12)	
Habit of exercise, n (%)				.570
Yes	529 (5.70)	547 (5.89)	561 (6.05)	
No	2564 (27.63)	2546 (27.44)	2533 (27.30)	
Alcohol consumption, n (%)				<.001
Heavy	69 (0.74)	115 (1.24)	126 (1.36)	
Moderate	220 (2.37)	301 (3.24)	305 (3.29)	
Light	318 (3.43)	366 (3.94)	367 (3.95)	
None	2486 (26.79)	2311 (24.90)	2296 (24.7)	
Smoking status, n (%)				<.001
Current	536 (5.78)	736 (7.93)	816 (8.79)	
Past	393 (4.23)	627 (6.76)	765 (8.24)	
Never	2164 (23.32)	1730 (18.64)	1513 (16.30)	
TG/HDL-C^a^ ratio, n (range)	0.79 (0.11-11.35)	1.16 (0.13-15.48)	1.89 (0.19-26.73)	<.001
Triglyceride-glucose index, mean (SD)	7.68 (0.56)	8.02 (0.57)	8.42 (0.60)	<.001
Metabolic score for insulin resistance, mean (SD)	27.45 (4.55)	30.63 (5.47)	35.52 (6.75)	<.001
FHTHWA index, mean (SD)	0.20 (0.12)	1.01 (0.43)	12.60 (24.44)	<.001
Follow-up duration, years, mean (SD)	6.79 (3.86)	5.88 (3.79)	5.47 (3.57)	<.001
Progressed to diabetes, n (%)				<.001
Yes	4 (0.04)	19 (0.20)	190 (2.05)	
No	3089 (33.29)	3074 (33.13)	2904 (31.29)	

a TG/HDL-C: triglyceride to high-density lipoprotein cholesterol.

For the validation set, patients with increased FHTHWA index levels tended to be of advanced age and displayed characteristics indicative of fatty liver, with higher BMI, WC, ALT, AST, body weight, GGT, TC, TG, HbA_1c_, FPG, SBP, DBP, alcohol consumption, smoking, TG/HDLC ratio, TyG index, and METS-IR, as well as low HDL-C levels (Table S4 in Multimedia Appendix 1; all *P*<.05).

### Predictive Value of the FHTHWA Index

**Figure 1.**
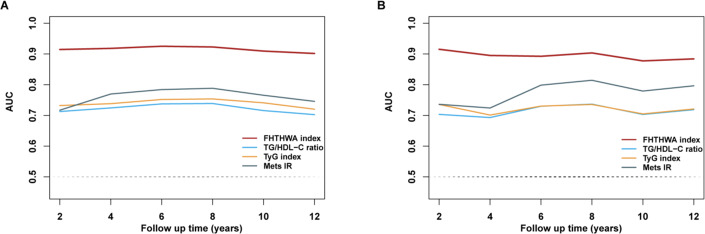
Time-dependent receiver operator characteristic curves of the FHTHWA index vary with time to predict the future risk of diabetes. (**A**) Area under the curve (AUC) comparison of the FHTHWA index, TG/HDL-C ratio, TyG index, and METS-IR in the training set. (**B**) AUC comparison of the FHTHWA index, TG/HDL-C ratio, TyG index, and METS-IR in the validation set. METS-IR: metabolic score for insulin resistance; TG/HDL-C: triglyceride to high-density lipoprotein cholesterol ratio; TyG index: triglyceride-glucose index.

Time-dependent ROC curves were utilized in order to assess the predictive efficacy of the FHTHWA index, as well as other newly proposed predictive parameters such as TG/HDL-C, the TyG index, and METS-IR, in relation to the risk of developing diabetes in the future. Figure 1A shows the AUC of the FHTHWA index and other novel predictive parameters over time for the training set. Overall, the baseline FHTHWA index demonstrated strong predictive capabilities for future diabetes occurrence at various time intervals. The AUCs of the FHTHWA index were higher compared with the AUCs of the TG/HDL-C, TyG index, and METS-IR during the 12-year follow-up. Figure 1B shows the AUC of the FHTHWA index and other novel predictive parameters over time for the validation set. Overall, the baseline FHTHWA index demonstrated strong predictive capability for future diabetes occurrence at various time intervals. Furthermore, the FHTHWA index exhibited greater AUC values compared to the TG/HDL-C, TyG index, and METS-IR at all time points.

### Relationship of the FHTHWA Index With Diabetes Risk

**Figure 2.**
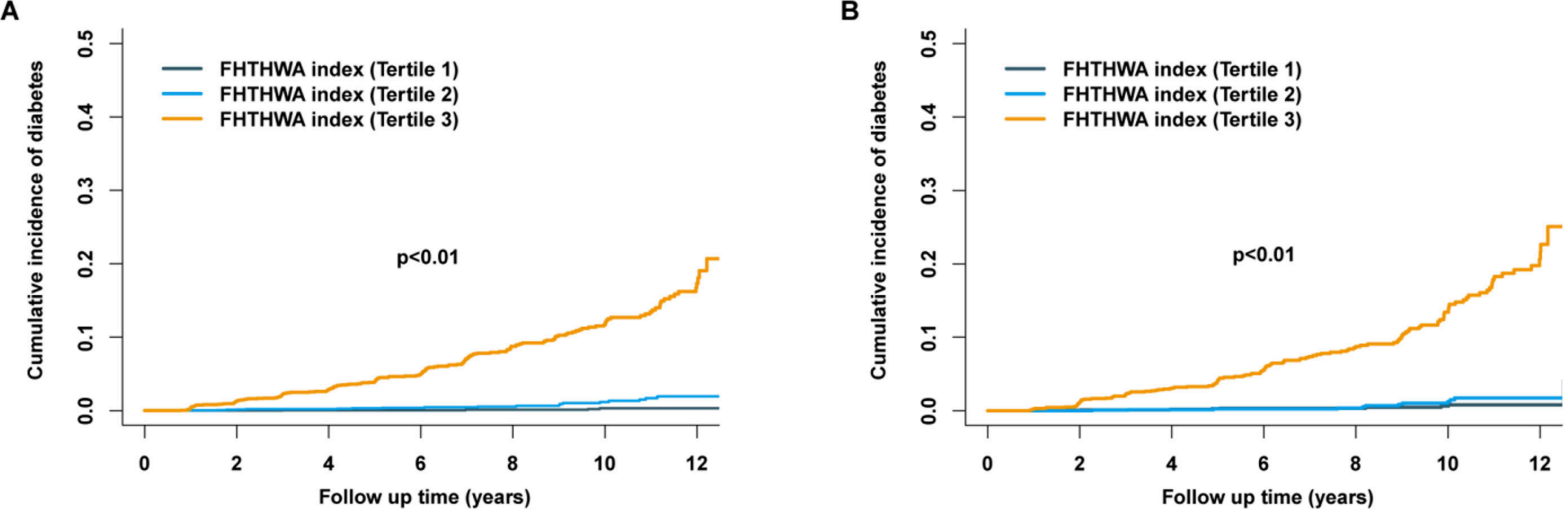
Weighted Kaplan-Meier curves for diabetes incidence by the baseline FHTHWA index strata. (**A**) Diabetes risk across the FHTHWA index strata in the training set exhibited divergence over time. (**B**) Diabetes risk across the FHTHWA index strata in the validation set exhibited divergence over time.

In the training set, 213 (1.38%) cases progressed to diabetes during the follow-up period. From quartile 1 to quartile 3 of the FHTHWA index, the diabetes rate increased gradually from 0.04% to 2.05% (Table 1). After multivariate adjustment, including sex, consumption of alcohol, smoking status, habit of exercise, BMI, fatty liver, ALT, AST, and GGT, an elevated FHTHWA index was markedly correlated with diabetes risk (Table 2). Multivariate adjusted hazard ratios and 95% CIs from the lowest to the highest FHTHWA index categories (tertile 1, tertile 2, and tertile 3) were 1.00 (reference), 4.509 (1.520‐13.373), and 32.141 (11.545‐89.476), respectively, for the incidence of diabetes (*P*<.001). The analysis of weighted Kaplan-Meier curves demonstrated significant dose-response patterns within the FHTHWA index strata in relation to the incidence of diabetes. These trends persisted even after a 12-year follow-up period. Furthermore, the risk of developing diabetes across the FHTHWA index strata displayed a noticeable divergence over time (Figure 2A).

**Table 2.** Hazard ratios and corresponding 95% CIs for diabetes incidence according to the FHTHWA index among the population without diabetes in the training set.

	FHTHWA index			*P* trend	Per unit increment in the FHTHWA index
	Tertile 1	Tertile 2	Tertile 3		
Number of cases of diabetes mellitus/total	4/3093	19/3093	190/3094		
Crude	1.000 (ref.)	5.846 (1.988‐17.158)	66.098 (24.542‐178.020)	<.001	1.019 (1.018‐1.021)
Model 1^a^	1.000 (ref.)	5.720 (1.914‐16.856)	64.129 (23.679‐173.677)	<.001	1.020 (1.018‐1.022)
Model 2^b^	1.000 (ref.)	4.975 (1.684‐14.695)	39.714 (14.459‐109.084)	<.001	1.017 (1.015‐1.019)

a Model 1: adjusted for sex, alcohol consumption, smoking status, and habit of exercise.

b Model 2: further adjusted (from Model 1) for BMI, fatty liver, ALT, AST, and GGT.

In the validation set, 160 (2.59%) cases progressed to diabetes during the follow-up period. From quartile 1 to quartile 3 of the FHTHWA index, the diabetes rate increased gradually from 0.16% to 2.25% (Table S4 in Multimedia Appendix 1). After multivariate adjustment, including sex, consumption of alcohol, smoking status, habit of exercise, BMI, fatty liver, ALT, AST, GGT, TC, TG, SBP, DBP, TyG, TG/HDL-C ratio, and METS-IR, an elevated FHTHWA index was markedly correlated with diabetes risk (Table S5 in Multimedia Appendix 1). Multivariate adjusted hazard ratios and 95% CIs from the lowest to the highest FHTHWA index categories (tertile 1, tertile 2, and tertile 3) were 1.00 (reference), 1.010 (0.419‐2.435), and 9.924 (4.830‐20.392), respectively, for the incidence of diabetes (*P*<.001). The utilization of weighted Kaplan-Meier curves demonstrated significant dose-response patterns across different strata of the FHTHWA index in relation to the incidence of diabetes, even after a follow-up period of 12 years. Moreover, the risk of developing diabetes across the FHTHWA index strata exhibited a noticeable divergence over time (Figure 2B).

### Stratification Analyses

In the stratified analyses of the dose-response association between FHTHWA index levels and diabetes risk, participants were stratified by sex (male or female), habit of exercise (yes or no), BMI (<25 kg/m^2^ or ≥25 kg/m^2^), current smoker (yes or no), and current alcohol use (yes or no). In the training set, we discovered that a high FHTHWA index was linked with a higher diabetes risk, contrasted with the reference group in all subgroups (Table 3). In the validation set, the results were the same as our main results (Table S6 in Multimedia Appendix 1).

**Table 3.** Stratified analyses of the associations (hazard ratios, 95% CIs) between the FHTHWA index and diabetes incidence at the end of follow-up among people with diabetes in the training set.

Variables		Hazard ratio (95% CI)^a^	
	Event, n/N	Unadjusted model	Multivariable-adjusted model^a^
Sex			
Male	157/5088	1.019 (1.017‐1.021)	1.020 (1.017‐1.023)
Female	56/4192	1.019 (1.017‐1.022)	1.013 (1.009‐1.017)
Habit of exercise			
Yes	35/1637	1.036 (1.030‐1.042)	1.028 (1.019‐1.037)
No	178/7643	1.019 (1.017‐1.020)	1.015 (1.013‐1.018)
BMI, kg/m^2^			
<25	113/7752	1.020 (1.018‐1.023)	1.020 (1.015‐1.025)
≥25	100/1528	1.016 (1.013‐1.018)	1.015 (1.012‐1.017)
Current smoker			
Yes	88/2088	1.032 (1.027‐1.036)	1.030 (1.024‐1.036)
No	125/7192	1.019 (1.017‐1.020)	1.015 (1.012‐1.017)
Current alcohol use			
Yes	56/2187	1.017 (1.014‐1.020)	1.024 (1.018‐1.030)
No	157/7093	1.020 (1.018‐1.022)	1.014 (1.012‐1.017)

a Hazard ratios (95% CIs) were assessed using weighted Cox proportional regression that was fully adjusted except for the stratification factor.

## Discussion

### Study Findings and Comparison With Previous Works

Diabetes mellitus can lead to a range of complications that result in significant psychological and physical distress for individuals who carry the disease. Moreover, this places a substantial strain on the healthcare systems [24,25]. Nevertheless, the early detection of this condition is often hindered due to the absence of distinct symptoms and a limited focus on public healthcare. Early identification of the population with high risk for diabetes might help prevent the onset of diabetes or delay its progression [26]. In this study, we developed and validated a robust parameter, named the FHTHWA index, to forecast the development of the future diabetes likelihood depending on a comprehensive longitudinal cohort study of the Japanese physical examination population. Our results revealed that the higher the FHTHWA index, the higher the risk of developing diabetes. The correlation exhibited independence from conventional risk factors, such as sex, alcohol consumption, smoking status, habit of exercise, BMI, fatty liver, SBP, DBP, TyG, TG/HDL-C ratio, and laboratory parameters.

The early identification of individuals at high risk for diabetes is crucial for implementing preventive strategies and mitigating the burden of this disease. A growth of risk assessment tools for type 2 diabetes mellitus has been observed on a global scale, such as the ADA risk score [16], the Canadian Diabetes Risk Score (CANRISK) [27], the Finnish Diabetes Risk Score (FINDRISC) [15], the Indian Diabetes Risk Score (IDRS) [28], and Indonesian Undiagnosed Diabetes Mellitus (UDDM) scoring system [29]. Both the ADA and CANRISK tools have demonstrated efficacy in identifying individuals at risk within their respective communities; nevertheless, these products were specifically created to cater to the diverse populations residing in the United States and Canada, respectively. The validation of the IDRS took place in a low- to middle-income country. The IDRS is a valuable screening tool specifically for the mono-ethnic Indian population [28]. The scoring system originated in Indonesia. However, its accuracy has not been assessed through prospective evaluation [29]. Most of the predictive indices are genetic, demographic characteristics, and clinical parameters. With the intensive study of the pathogenesis of diabetes, many studies have begun to identify the genetic associations associated with the onset of diabetes. These studies not only reveal the genetic basis of diabetes but also provide new perspectives for understanding its pathogenesis. For example, recent genome-wide association studies identified multiple genetic variants associated with type 2 diabetes that may affect insulin secretion and islet β-cell function [30]. Furthermore, studies show that certain genetic variants are strongly associated with the age of onset of diabetes, and younger patients with the onset of diabetes often face a higher risk of cardiovascular disease and all-cause mortality [31]. The effects of genetic variation also vary in different populations. For example, studies in East Asian populations found that certain genetic variants associated with type 2 diabetes mellitus showed different effects in this population, underscoring the importance of genetic studies in different ethnic groups [32]. Moreover, studies targeting monogenic diabetes have also revealed genetic mechanisms associated with autoimmunity that may play an important role in polygenic diabetes [33]. It is very important to consider incorporating genes into predictors in future studies.

Moreover, most tools are based on the general population in defined regions and may not be generalizable to populations from different regions [34,35]. Currently, there is still a lack of widely used diabetes risk tools for the Japanese population [36]. The participants in this study were selected from a longitudinal cohort study that specifically targeted those undergoing health check-ups. This study commenced in 1994 and involved the continuous recruitment of individuals who underwent health examinations at Murakami Memorial Hospital. Thus, the study population typically included Japanese or Eastern Asian populations. The genetic constitution and lifestyle of Asian populations are different from those of European or American populations. Thus, the FHTHWA index might be suitable for the Japanese general population and other East Asian populations.

Insulin resistance (IR) is a medical condition characterized by reduced cellular responsiveness to insulin, independent of hyperinsulinemia [37]. IR serves as the primary pathophysiological mechanism underlying the developing type 2 diabetes mellitus, and it has the potential to manifest in individuals up to 2 decades prior to the diagnosis [38]. IR serves as a pivotal indicator of the imminent onset of diabetes. Evidence indicates that visceral adiposity indexes, such as BMI and WC, possess the capacity to effectively detect IR [39,40]. Moreover, evidence supports the effectiveness of classic lipid indices, including TC, TG, HDL-C, and LDL-C, in diagnosing IR [41,42]. However, the predictive capacities of these factors are limited [8]. Recently, the TyG index and TG/HDL-C have been recommended as diabetes risk indicators, both of which have better performance than traditional indicators (BMI, WC, TC, TG, HDL-C, and LDL-C) [43,44]. In this study, we performed a comparison of AUC values by time-dependent ROC analyses between the FHTHWA index, TyG index, TG/HDL-C, and METS-IR. It was found that the FHTHWA index had high accuracy, with the AUC value greater than 0.9 during the more than 12 years of follow-up. However, the AUC values of the TyG index, TG/HDL-C, and METS-IR mostly ranged from 0.7 to 0.8. Thus, the novel index has a higher prediction accuracy than the traditional parameters.

### Study Strengths

This study’s primary strengths include the large sample size of 15464 individuals ranging from 18‐79 years of age, who underwent thorough examination and were subsequently monitored over an extended duration of up to 13 years. This sample was representative of the entire nation, enabling the findings to be extrapolated to the broader Japanese or East Asian populations.

### Study Limitations

The present study has certain limitations. First, the primary focus of this study was the onset of diabetes as the outcome of interest. Nevertheless, it is important to acknowledge that the diagnostic criteria utilized in this investigation did not include the participants’ 2-hour postprandial blood glucose level. This has the potential to result in an underestimation of the incidence of diabetes. Second, due to the utilization of the NAGALA database, it was impossible to eliminate self-reported characteristics, resulting in a potential reporting bias. Consequently, the findings of this study can only be generalized to the Japanese or East Asian population. It is crucial to determine the degree to which these findings can be extrapolated to heterogeneous populations. Third, it is important to note that the primary outcome measure of this study is the occurrence of new-onset diabetes events. However, it is noteworthy to emphasize that the dataset utilized in this study does not include information on death events that may have occurred during the follow-up period. This limitation introduces a potential competing risk that could impact the findings of the current research.

### Conclusion

The research has successfully developed the FHTHWA index, which demonstrates a high degree of accuracy in predicting the future risk of diabetes within the Japanese population without diabetes. This index is particularly valuable for clinical practitioners in Japan, as it is recommended for regular use in assessing the risk of diabetes and monitoring therapeutic progress among the Japanese population.

## Supplementary material

10.2196/64992 Multimedia Appendix 1 Supplementary files.

## Abbreviations

ADA: American Diabetes Association
ALT: alanine aminotransferase
AST: aspartate aminotransferase
AUC: area under the curve
CANRISK: Canadian Diabetes Risk Score
DBP: diastolic blood pressure
FINDRISC: Finnish Diabetes Risk Score
FPG: fasting plasma glucose
GGT: gamma-glutamyl transferase
HDL-C: high-density lipoprotein cholesterol
IDF: International Diabetes Federation
IDRS: Indian diabetes risk score
IR: insulin resistance
LASSO: least absolute shrinkage and selection operator
LDL-C: low-density lipoprotein cholesterol
METS-IR: metabolic score for insulin resistance
NAGALA: NAfld [nonalcoholic fatty liver disease] in the Gifu Area, Longitudinal Analysis
NHANES: National Health and Nutrition Examination Survey
ROC: receiver operating characteristic
SBP: systolic blood pressure
TC: total cholesterol
TC/HDL-C: total cholesterol to high-density lipoprotein cholesterol ratio
TG: triglyceride
TG/HDL-C: triglyceride to high-density lipoprotein cholesterol ratio
TyG index: triglyceride-glucose index
UDDM: Indonesian Undiagnosed Diabetes Mellitus
WC: waist circumference
