# Supplementary material for: Use of the FHTHWA Index as a Novel Approach for Predicting the Incidence of Diabetes in a Japanese Population Without Diabetes: Data Analysis Study
Source: JMIR Med Inform. 2025 Jan 28;13:e64992. doi: 10.2196/64992 (PMC11793195; doi:10.2196/64992)
Supplement: Multimedia Appendix 1 [file medinform-v13-e64992-s001.docx]

| **Table S1. Baseline demographic, lifestyle, and laboratory characteristics in participants (Training vs Validation Set).** | | | | |
| --- | --- | --- | --- | --- |
| Characteristics | Train (n=9280) | Validation (n=6184) | Total (n=15464) | P value |
| Gender |  |  |  | 0.350 |
| Male | 5088 (32.90%) | 3342 (21.61%) | 8430 (54.51%) |  |
| Female | 4192 (27.11%) | 2842 (18.38%) | 7034 (45.49%) |  |
| Age, years | 42 (18, 79) | 42 (20, 79) | 42 (18, 79) | 0.760 |
| Body Weight, kg | 60.59 ± 11.60 | 60.71 ± 11.64 | 60.64 ± 11.62 | 0.415 |
| BMI, kg/m^2^ | 22.11 ± 3.14 | 22.13 ± 3.11 | 22.12 ± 3.13 | 0.617 |
| WC, cm | 76.51 ± 9.08 | 76.42 ± 9.15 | 76.47 ± 9.11 | 0.320 |
| ALT, IU/L | 17 (4, 149) | 17 (2.00, 856.00) | 17 (2, 856) | 0.430 |
| AST, IU/L | 17 (3,150) | 17 (4, 590) | 17 (3, 590) | 0.129 |
| GGT, IU/L | 15 (3,399) | 15 (3, 322) | 15 (3, 399) | 0.631 |
| HDL-C, mmol/L | 1.40 (0.00, 3.49) | 1.42 (0.00, 4.49) | 1.41 (0.00, 4.49) | <0.05 |
| TC, mmol/L | 5.12 ± 0.86 | 5.14 ± 0.86 | 5.13 ± 0.86 | 0.952 |
| TG, mmol/L | 0.73 (0.07, 7.69) | 0.73 (0.08, 10.27) | 0.73 (0.07, 10.27) | 0.291 |
| HbA1c, % | 5.17 ± 0.32 | 5.18 ± 0.32 | 5.17 ± 0.32 | 0.066 |
| Fatty liver |  |  |  | 0.980 |
| Yes | 1646 (10.64%) | 1095 (7.08%) | 2741 (17.73%) |  |
| No | 7634 (49.37%) | 5089 (32.91%) | 12723 (82.27%) |  |
| Habit of exercise |  |  |  | 0.640 |
| Yes | 1637 (10.59%) | 1072 (6.93%) | 2709 (17.52%) |  |
| No | 7643 (49.42%) | 5112 (33.06%) | 12755 (82.48%) |  |
| Alcohol consumption |  |  |  | 0.570 |
| Heavy | 310 (2.00%) | 231 (1.49%) | 541 (3.50%) |  |
| Moderate | 826 (5.34%) | 534 (3.45%) | 1360 (8.79%) |  |
| Light | 1051 (6.80%) | 707 (4.57%) | 1758 (11.37%) |  |
| Non | 7093 (45.87%) | 4712 (30.47%) | 11805 (76.34%) |  |
| Smoking status |  |  |  | 0.850 |
| Current | 2088 (13.50%) | 1393 (9.01%) | 3481 (22.51%) |  |
| Past | 1785 (11.54%) | 1167 (7.55%) | 2952 (19.09%) |  |
| Never | 5407 (34.97%) | 3624 (23.44%) | 9031 (58.40%) |  |
| FPG, mmol/L | 5.16 ± 0.41 | 5.16 ± 0.41 | 5.16 ± 0.41 | 0.780 |
| SBP, mmHg | 114.57 ± 15.01 | 114.40 ± 14.92 | 114.50 ± 14.97 | 0.126 |
| DBP, mmHg | 71.65 ± 10.52 | 71.47 ± 10.48 | 71.58 ± 10.50 | 0.082 |
| TG/HDL-C ratio | 1.17 (0.11, 26.73) | 1.15 (0.07, 37.92) | 1.17 (0.07, 37.92) | 0.165 |
| TyG index | 8.04 ± 0.65 | 8.03 ± 0.64 | 8.03 ± 0.65 | 0.565 |
| Mets-IR | 31.20 ± 6.56 | 31.11 ± 6.40 | 31.16 ± 6.50 | 0.105 |
| Follow up duration, years | 6.04 ± 3.78 | 6.06 ± 3.78 | 6.05 ± 3.78 | 0.555 |
| Progressed to diabetes |  |  |  | 0.270 |
| Yes | 213 (1.38%) | 160 (1.03%) | 373 (2.41%) |  |
| No | 9067 (58.63%) | 6024 (38.95%) | 15091 (97.59%) |  |

| **Table S2. Univariate Cox regression analysis to screen candidate [metabolic index](javascript:;).** | | | |
| --- | --- | --- | --- |
| Variables | Hazard Ratio | 95% Confidence Interval | P value |
| Age | 1.059 | 1.043-1.076 | <0.01 |
| BMI | 1.225 | 1.193-1.258 | <0.01 |
| WC | 1.088 | 1.075-1.101 | <0.01 |
| HDL-c | 0.167 | 0.111-0.251 | <0.01 |
| TC | 1.528 | 1.334-1.751 | <0.01 |
| TG | 1.952 | 1.762-2.162 | <0.01 |
| HbA1c | 72.797 | 47.447-111.692 | <0.01 |
| FPG | 22.541 | 15.171-33.493 | <0.01 |

| **Table S3. Multivariate Cox regression analyses to build a [metabolic parameters](javascript:;) based risk index.** | | | | |
| --- | --- | --- | --- | --- |
| Variables | Coefficient | Hazard Ratio | 95% Confidence Interval | P value |
| Age | 0.016 | 1.017 | 1.000-1.034 | 0.051 |
| WC | 0.044 | 1.045 | 1.029-1.060 | <0.01 |
| HDL-c | -0.454 | 0.635 | 0.390-1.033 | 0.067 |
| TG | 0.216 | 1.241 | 1.056-1.459 | <0.01 |
| HbA1c | 3.298 | 27.072 | 16.976-43.171 | <0.01 |
| FPG | 1.525 | 4.594 | 2.979-7.085 | <0.01 |

| **Table S4. Baseline characteristics of the study population by tertiles of FHTHWA index in validation set** | | | | |
| --- | --- | --- | --- | --- |
| Characteristics | Tertile 1 (n=2061) | Tertile 2 (n=2061) | Tertile 3 (n=2062) | P value |
| Gender |  |  |  | <0.01 |
| Male | 694 (11.22%) | 1178 (19.05%) | 1470 (23.77%) |  |
| Female | 1367 (22.11%) | 883 (14.28%) | 592 (9.57%) |  |
| Age, years | 40 (20, 75) | 42 (20, 77) | 47 (24, 79) | <0.01 |
| BMI, kg/m2 | 20.44 ± 2.32 | 21.97 ± 2.64 | 23.97 ± 3.22 | <0.01 |
| WC, cm | 70.63 ± 7.13 | 76.33 ± 7.82 | 82.29 ± 8.45 | <0.01 |
| ALT, IU/L | 14 (2, 856) | 17 (2, 116) | 20 (5,224) | <0.01 |
| AST, IU/L | 16 (6, 590) | 17 (6, 122) | 19 (4,160) | <0.01 |
| Body Weight, kg | 54.37 ± 9.04 | 60.72 ± 10.38 | 67.03 ± 11.71 | <0.01 |
| GGT, IU/L | 12 (3, 322) | 15 (4, 242) | 19 (5, 249) | <0.01 |
| HDL-C, mmol/L | 1.58 (0.43,3.43) | 1.43 (0.52,4.49) | 1.25 (0, 2.90) | <0.01 |
| TC, mmol/L | 4.88 ± 0.78 | 5.12 ± 0.84 | 5.42 ± 0.88 | <0.01 |
| TG, mmol/L | 0.55 (0.08,4.27) | 0.73 (0.10,6.20) | 1.02 (0.12,10.27) | <0.01 |
| HbA1c, % | 4.91 ± 0.22 | 5.16 ± 0.21 | 5.46 ± 0.24 | <0.01 |
| FPG, mmol/L | 4.84 ± 0.31 | 5.16 ± 0.31 | 5.50 ± 0.31 | <0.01 |
| SBP, mmHg | 108.21 ± 13.19 | 114.38 ± 13.85 | 120.59 ± 15.03 | <0.01 |
| DBP, mmHg | 67.39 ± 9.19 | 71.43 ± 9.94 | 75.60 ± 10.62 | <0.01 |
| Fatty liver |  |  |  | <0.01 |
| Yes | 75 (1.21%) | 276 (4.46%) | 744 (12.03%) |  |
| No | 1986 (32.12%) | 1785 (28.86%) | 1318 (21.31%) |  |
| Habit of exercise |  |  |  | 0.950 |
| Yes | 353 (5.71%) | 358 (5.79%) | 361 (5.84%) |  |
| No | 1708 (27.62%) | 1703 (27.54%) | 1701 (27.51%) |  |
| Alcohol consumption |  |  |  | <0.01 |
| Heavy | 52 (0.84%) | 74 (1.20%) | 105 (1.70%) |  |
| Moderate | 165 (2.67%) | 173 (2.80%) | 196 (3.17%) |  |
| Light | 216 (3.49%) | 251 (4.06%) | 240 (3.88%) |  |
| Non | 1628 (26.33%) | 1563 (25.27%) | 1521 (24.60%) |  |
| Smoking status |  |  |  | <0.01 |
| Current | 357 (5.77%) | 479 (7.75%) | 557 (9.01%) |  |
| Past | 275 (4.45%) | 380 (6.14%) | 512 (8.28%) |  |
| Never | 1429 (23.11%) | 1202 (19.44%) | 993 (16.06%) |  |
| TG/HDL-C ratio | 0.79 (0.07,12.23) | 1.15 (0.12,13.66) | 1.83 (0.21,37.92) |  |
| TyG index | 7.67 ± 0.53 | 8.01 ± 0.56 | 8.41 ± 0.60 | <0.01 |
| Mets-IR | 27.25 ± 4.38 | 30.68 ± 5.32 | 35.42 ± 6.47 | <0.01 |
| FHTHWA Index | 0.21 ± 0.12 | 1.04 ± 0.45 | 12.71 ± 28.77 | <0.01 |
| Follow up duration, years | 6.82 ± 3.83 | 5.93 ± 3.81 | 5.42 ± 3.57 | <0.01 |
| Progressed to diabetes |  |  |  | <0.01 |
| Yes | 10 (0.16%) | 11 (0.18%) | 139 (2.25%) |  |
| No | 2051 (33.17%) | 2050 (33.15%) | 1923 (31.10%) |  |

| **Table S5. HR (95% CIs) for diabetes incedence according to FHTHWA index among non-diabetic population in the validation set.** | | | | | |
| --- | --- | --- | --- | --- | --- |
|  | FHTHWA index | | | p trend | Per one-unit increment FHTHWA index |
|  | Tertile 1 | Tertile 2 | Tertile 3 |  |  |
| Number of DM /total | 10/2061 | 11/2061 | 139/2062 |  |  |
| Crude | 1.000 (ref.) | 1.372 (0.582-3.231) | 20.518 (10.758-39.032) | <0.01 | 1.010 (1.009-1.011) |
| Model 1 | 1.000 (ref.) | 1.233 (0.520-2.922) | 17.617 (9.095-34.124) | <0.01 | 1.009 (1.008-1.010) |
| Model 2 | 1.000 (ref.) | 1.027 (0.430-2.452) | 11.045 (5.556-21.958) | <0.01 | 1.007 (1.005-1.008) |
| Model 3 | 1.000 (ref.) | 1.010 (0.419-2.435) | 9.924 (4.830-20.392) | <0.01 | 1.005 (1.004-1.007) |
| Model 1: adjusted for gender, alcohol consumption, smoking status, and habit of exercise. | | | | | |
| Model 2: further adjusted (from Model 1) for BMI, fatty liver, ALT, AST, and GGT. | | | | | |
| Model 3: further adjusted (from Model 2) for TC, TG, SBP, DBP, TyG, TG/HDL-c ratio, and Mets IR. | | | | | |

| **Table S6. Stratified analyses of the associations (hazard ratios, 95% CIs) between FHTHWA index and Diabetes incidence at the End of Follow-up among diabetes in validation set.** | | | |
| --- | --- | --- | --- |
| Variables | Event, n/total | Hazard ratio (95% confdence interval) | |
|  |  | Unadjusted model | Multivariable-adjusted model |
| Age, years | | | |
| <60 | 147/5907 | 1.010 (1.009-1.011) | 1.006 (1.004-1.007) |
| ≥60 | 13/277 | 1.022 (1.012-1.031) | 1.065 (1.034-1.097) |
| Sex |  |  |  |
| Male | 129/3342 | 1.009 (1.008-1.010) | 1.050 (1.027-1.075) |
| Female | 31/2842 | 1.038 (1.028-1.049) | 1.026 (1.011-1.041) |
| Habit of exercise | | | |
| Yes | 16/1072 | 1.021 (1.013-1.030) | 1.025 (1.012-1.039) |
| No | 144/5122 | 1.010 (1.009-1.011) | 1.005 (1.003-1.007) |
| BMI, kg/m2 | | | |
| <25 | 88/5188 | 1.039 (1.034-1.044) | 1.033 (1.025-1.041) |
| ≥25 | 72/996 | 1.008 (1.006-1.009) | 1.006 (1.004-1.008) |
| Current smoker | | | |
| Yes | 63/1393 | 1.008 (1.007-1.010) | 1.005 (1.003-1.008) |
| No | 97/4791 | 1.024 (1.021-1.027) | 1.018 (1.013-1.022) |
| Current alcohol use | | | |
| Yes | 51/1472 | 1.041 (1.034-1.047) | 1.030 (1.021-1.040) |
| No | 109/4712 | 1.010 (1.009-1.011) | 1.006 (1.004-1.008) |
| HRs (95%CI) were assessed using weighted Cox proportional regression fully adjusted except for stratification factor. | | | |


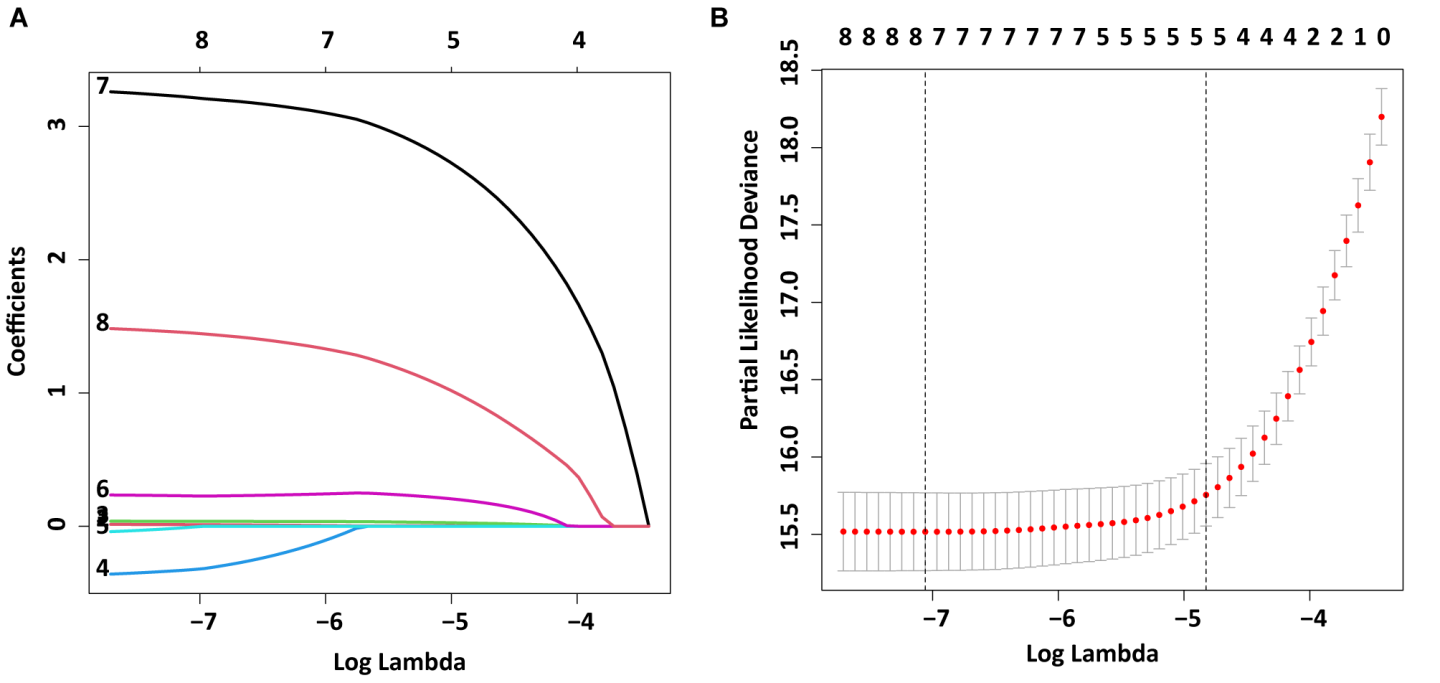


**Figure S1:** Lasso analysis was used to remove excessively associated factors, and 8 factors were identified as being significant.
